# Developing customized NIRS-EEG for infant sleep research: methodological considerations

**DOI:** 10.1117/1.NPh.10.3.035010

**Published:** 2023-09-25

**Authors:** Louisa K. Gossé, Paola Pinti, Frank Wiesemann, Clare E. Elwell, Emily J. H. Jones

**Affiliations:** a Birkbeck, University of London, Centre for Brain and Cognitive Development, London, United Kingdom; b Research and Development, Procter & Gamble, Schwalbach am Taunus, Germany; c University College London, Department of Medical Physics and Biomedical Engineering, Biomedical Optics Research Laboratory, London, United Kingdom

**Keywords:** infant, sleep, functional near-infrared spectroscopy, EEG, data quality, wearable neuroimaging

## Abstract

**Significance:**

Studies using simultaneous functional near-infrared spectroscopy (fNIRS)-electroencephalography (EEG) during natural sleep in infancy are rare. Developments for combined fNIRS-EEG for sleep research that ensure optimal comfort as well as good coupling and data quality are needed.

**Aim:**

We describe the steps toward developing a comfortable, wearable NIRS-EEG headgear adapted specifically for sleeping infants ages 5 to 9 months and present the experimental procedures and data quality to conduct infant sleep research using combined fNIRS-EEG.

**Approach:**

N=49 5- to 9-month-old infants participated. In phase 1, N=26 (10 = slept) participated using the non-wearable version of the NIRS-EEG headgear with 13-channel-wearable EEG and 39-channel fiber-based NIRS. In phase 2, N=23 infants (21 = slept) participated with the wireless version of the headgear with 20-channel-wearable EEG and 47-channel wearable NIRS. We used QT-NIRS to assess the NIRS data quality based on the good time window percentage, included channels, nap duration, and valid EEG percentage.

**Results:**

The infant nap rate during phase 1 was ∼40% (45% valid EEG data) and increased to 90% during phase 2 (100% valid EEG data). Infants slept significantly longer with the wearable system than the non-wearable system. However, there were more included good channels based on QT-NIRS in study phase 1 (61%) than phase 2 (50%), though this difference was not statistically significant.

**Conclusions:**

We demonstrated the usability of an integrated NIRS-EEG headgear during natural infant sleep with both non-wearable and wearable NIRS systems. The wearable NIRS-EEG headgear represents a good compromise between data quality, opportunities of applications (home visits and toddlers), and experiment success (infants’ comfort, longer sleep duration, and opportunities for caregiver–child interaction).

## 1 Introduction

### 1.1 fNIRS in Sleep Research

Functional near-infrared spectroscopy (fNIRS) is primarily used in awake populations, but there is increasing interest in its potential for sleep research. Sleep has great potential to be a new use case for fNIRS, but the development of new methodologies (both analytically and practically) is crucial. For example, some adult studies have shown that different sleep stages showed differing oxygenation levels as assessed by fNIRS^1^ and that oxygenated (HbO) and deoxygenated (HbR) hemoglobin desynchronized before sleep-wake transitions.^2^ Transitions between sleep stages were accompanied by an increase in HbR when transitioning into deeper sleep stages and an increase in HbO in the transitions to lighter sleep phases and wake.^3^ Similar results were found by Metz et al. in adolescents.^4^ fNIRS has also been used in neonatal studies for sleep research specifically and in developmental functional connectivity studies, which are often done on sleeping infants as the data are easier to acquire (e.g., Refs. 5–7). For example, Lee et al. compared fNIRS-measured resting state connectivity in active and quiet sleep states (i.e., precursors of developmentally later emerging rapid eye movement (REM) and non-rapid eye movement (NREM) sleep) in healthy neonates.^8^ They showed more long-range connectivity during active sleep and more short-range connectivity during quiet sleep, in line with the results found in adult studies that connectivity patterns differ depending on sleep stage.^9^ The first study investigating functional connectivity using fNIRS and EEG during sleep states was conducted on adults and revealed that, with deeper sleep (stage N2 compared with N1 sleep), the network connectivity decreased, which the authors explained as mirroring the reduced response of a sleeping adult to their environment.^9^ In summary, using fNIRS during sleep can provide useful information about the physiology and function of sleep (i.e., how different sleep stages might contribute to the development of connectivity) that might otherwise not be captured.

### 1.2 fNIRS for Infant Sleep Research—Why Should We Combine it with EEG?

Sleep in infancy has been shown to be related to infant’s concurrent and subsequent development,^10^^–^^15^ though the question of directionality has yet to be answered. Recent evidence suggests that in studying the sleeping infant’s brain this relationship can be further disentangled.^16^ Studies have revealed a benefit of napping after an experimental task for learning artificial languages and language in general,^17^^–^^19^ generalization of learned information,^10^^,^^11^ or performance on a motor (memory) task,^20^^–^^22^ to name a few examples. In conclusion, napping after learning new information is essential for infants to commit information into short-^23^ and long-term memory^24^^,^^25^ with cascading effects on development by influencing skill acquisition. These studies in the infant sleep literature use EEG markers of sleep that include examining the frequency and density of sleep spindles and the proportion of slow wave activity/slow wave sleep (SWA/SWS).^16^

Recently, researchers have also found that connectivity patterns are indicative of developmental status. Generally, the pattern arising across development (from infancy to adulthood) is one of local connectivity, i.e., spatially close neuronal groups, to global, i.e., spatially distant neuronal groups.^26^ Brain plasticity, the brain’s ability to forge new connections and prune unnecessary connections, is integral to forming connectivity in development. As proposed by major theories in sleep research, e.g., the synaptic homeostasis hypothesis, plasticity is crucially dependent on sleep.^27^^,^^28^ To use EEG for comprehensive connectivity analyses, many electrodes are needed to perform source-localization and obtain accurate localization results. This poses problems when requiring participants to sleep as extensive coverage with electrodes makes wearing the cap uncomfortable. fNIRS could be the solution to understanding how sleep and development are related while reducing the number of sensors needed and obtaining more accurate spatial localization. Moreover, as some studies have shown sleep stage transitions to be associated with changes in hemodynamic activity in the brain in adults,^3^^,^^29^ it is crucial to investigating neurovascular coupling during sleep in infants. Thus, combining fNIRS with EEG is important from both empirical and theoretical perspectives.

Though, to determine true sleep (awake versus asleep) and sleep stages (i.e., NREM versus REM sleep) and link existing markers of development during sleep (e.g., sleep spindles^16^), measuring some EEG channels is indispensable at the moment. Therefore, here we propose that the multimodal acquisition of EEG and fNIRS data can provide further insight into infants’ sleep and how infant sleep might be associated with development.

### 1.3 Representative Review of Studies Combining fNIRS and EEG in Infancy (Sleep) Research

The earliest studies on combined NIRS-EEG during sleep were conducted in hospital settings and in newborns. In pioneering work, Roche-Labarbe and colleagues combined measurement of 8 electrodes and temporal cortex NIRS measurements in a small sample (10 prematurely born neonates). They found that spontaneous neuronal activity in quiet sleep was associated/accompanied by an initial HbR increase, followed by a stronger increase in HbO. This early combined study of NIRS-EEG in sleeping very young infants proved that it is possible to record both methods at the same time in infants.^30^ Singh et al.^31^ also used diffuse optical tomography (DOT) and EEG to investigate hemodynamics of seizures in N=1 neonate. Other studies with newborns looked at the development of speech characteristics and/or processing of other acoustic signals making use of EEG’s ability to detect fast temporal modulations and combining it with fNIRS’ ability for accurate localization.^32^^,^^33^

More recently, research focused on fNIRS-measured connectivity in newborns, with some combining EEG for the purpose of sleep measurements. For example, a recent PhD project examined how task-based EEG and fNIRS are related in awake infants in the first year of life.^34^ In addition, Lee et al. showed that there was more interhemispheric connectivity during active sleep than during quiet sleep in newborns and more intra-hemispheric connectivity during quiet sleep than during active sleep in a sample of N=20 neonates using a 69-channel fiber-based NIRS.^8^ Taga et al. showed overall higher connectivity during sleep compared with wake in infants of the first year of life and more global activity in sleep compared with wake in N=91 infants using a 48-channel fiber-based NIRS system but without collecting EEG data. These results are supported by prior research that suggested that certain sleep states, such as SWS, potentially facilitate memory consolidation by inducing cross-regional brain connectivity changes^35^ in adults. Furthermore, sleep deprivation was found to alter functional connectivity as measured by EEG and functional magnetic resonance imaging (fMRI) during subsequent sleep.^36^^,^^37^ Taken together, these studies imply that brain functional connectivity is fundamentally different depending on the sleep state. Therefore, investigating functional connectivity changes in the brain through hemodynamic-based measures has the potential to classify sleep stages similarly to traditional EEG assessments. These studies have been conducted either in newborns or adults, but this setup has yet to be tested in older infants.

Past research suggests that it is possible to combine fNIRS with EEG for sleep measurements, that connectivity patterns fluctuate across the duration of sleep in newborns and in adults, and that those fluctuations might reveal more information about and map onto underlying sleep stages. Moreover, prior research suggests that the measurement of fNIRS and EEG could potentially reveal important information about how investigating connectivity during sleep could provide insight into the relationship between sleep and development. However, method development using fNIRS-EEG for sleep research is still in its infancy. Although there has been work in sleep fNIRS-EEG for newborns, there is no such research for older infants or childhood. The age range of mid-late infancy shows substantive changes in both neurocognitive and sleep development. For example, sleep stages start to resemble the adult sleep patterns compared with newborns, and characteristics such as sleep spindles, which are crucial indicators of learning during sleep, have emerged in most infants over the course of the first months of life. Therefore, before functional connectivity can be investigated in relation to sleep markers, there is need for a feasibility study that lays the foundation for widespread use of this innovative technology for sleep research. The aim of the present study is to conduct this feasibility study and understand the practicalities and data yields in this age group.

### 1.4 Challenges of Conducting Combined NIRS-EEG Sleep Research in Infancy

Studying the sleeping infant’s brain comes with many challenges that adult sleep research, especially adult research including fNIRS technology, does not face. Below we discuss the challenges that we set to address with this proof-of-concept study, including headgear design and experimental protocols, which are described in Sec. 2 (“Methods”).

#### 1.4.1 Challenge 1: combination of NIRS-EEG for sleep research

The first challenge is to design a headgear that accommodates both fNIRS optodes and EEG electrodes without corrupting the quality of either data and to allow for data synchronization across both data streams. For example, adult studies have placed electrodes in between NIRS optodes to measure the hemodynamic signal across the same area as the electrical activity of the brain,^38^ though this approach could be challenging as the source–detector separation might prove too large for infant studies, thus corrupting accurate measurement. The placement of NIRS optodes and EEG electrodes alongside each other is especially challenging in infant headgears due to the limited space compared with larger adult caps. Moreover, as electrodes are very close to optodes, gel leakage from wet electrodes can lead to, in the worst case, corruption of the optodes and, in the best case, bad data quality. The headgear should enable the placement of EEG electrodes alongside the NIRS electrodes for the measurement of EEG activity to identify sleep stages and sleep stage markers, such as sleep spindles. The placement of electrodes on the midline is essential to be in accordance with common polysomnography (PSG) placement to identify, e.g., sleep spindles.^39^ PSG refers to the gold-standard systematic measurement of physiological markers (with EEG, electrooculogram, electromyography, respiration, and cardiac measures) to assess the sleep state.^40^ Sleep spindles occur frequently along the midline and are most easily identified there in PSG recordings, necessitating placement of electrodes in this location.^41^

#### 1.4.2 Challenge 2: comfort

A primary concern for sleep studies more so than awake studies is comfort. For the purpose of studying sleep, the primary goal should be comfort during wear time. This is especially true if the goal is to get infants to sleep in an unfamiliar environment such as a laboratory. Infants compared with newborns have lower sleep pressure, and physical discomfort or environmental disturbances, such as noise, might make falling asleep harder for them than for newborns. Therefore, the cap comfort is key. The cap will be worn for longer than in typical infant studies in which study durations rarely exceed half an hour. By contrast, infants may nap up to 3 hrs at a time.^42^ The NIRS optode pressure on the skin may be too much for long studies involving the sensitive infant skin. Any cap design must account for the increased duration and pressure that infants may exert by lying on the optodes. Identifying a way to reduce the amount of pressure that optodes as well as EEG electrodes put on the infant’s skin is key in the development of the headgear. Many current infant fNIRS headgear designs use non-breathable fabrics or silicon bands to keep source–detector separation constant across infants.^43^^,^^44^ These materials are not suitable for the extended duration of the sleep studies as they potentially cause the infant to sweat and become very warm and potentially deteriorate the EEG signal. Any headgear design ought to provide a solution for increasing infant comfort.

#### 1.4.3 Challenge 3: sleep environment

Though not unique to a combined NIRS-EEG sleep study, the setup of an infant sleep study is important to achieving successful data collection. In particular, as fNIRS and EEG require more equipment than one method alone, the environment and the steps in putting an infant to sleep must be carefully considered. Please see Sec. 1.2 in the Supplementary Material for more information on the practicalities of infant sleep studies. Ideally, any headgear should also allow for some movement of the infant while they are sleeping and even allow for parents to pick up the infant and soothe them should they wake up during the nap. Along with the comfort aspect in challenge 2, the environment (e.g., place where the infant sleeps, room setup, lighting, etc.) also must be comfortable, not just the headgear.

### 1.5 Current Study

Consequently, we have two main goals in this study. (1) We aim to develop a customized fNIRS-EEG headgear for use on sleeping infants ages 5 to 9 months that addresses the challenges described in Sec. 1.4, and (2) we aim to investigate the feasibility of using this system in sleeping infants aged 5 to 9 months old. To achieve these goals, in this paper, we discuss off-the-shelf NIRS and EEG systems adapted for combined use and integrated into a bespoke cap, present and set out new ways to overcome the above-mentioned challenges and create a suitable setup, and evaluate data quality (EEG and fNIRS) and attrition rates comparing different setups.

## 2 Methods

### 2.1 Development of the Headgear Version 1 and Version 2

Below we describe the development of the two customized NIRS-EEG headgear options developed for the purpose of infant sleep research. The Supplementary Material includes a detailed description of how the headgear in this study was constructed as well as a step-by-step guide on how to successfully conduct infant sleep studies using NIRS-EEG.

#### 2.1.1 Review of existing customized NIRS-EEG headgears in the infant literature

Recently, Uchitel and colleagues provided an exhaustive review of NIRS-EEG developments in the literature,^45^ and we refer the readers to their work and work by Wallois et al.^46^ for a detailed overview into this topic. However, as our paper focuses on infants, we briefly review studies with unique NIRS-EEG headgears.

As briefly touched upon above, the earliest NIRS-EEG studies are based in hospital settings with the goal of cot-side monitoring (prematurely born) newborns with neurological damage (e.g., stroke) via NIRS to monitor cerebral oxygenation and EEG to screen for seizure.^30^^,^^47^ More recent research has extended these approaches.^31^^,^^48^^–^^52^ Some studies used two separate headgears for each system, e.g., Ref. 52, which is convenient as a new cap design is not required. Others utilized customized opto-electrodes,^47^^,^^51^ which for labs unaffiliated with engineering departments represents a significant financial barrier. Finally, fiber-based NIRS and stationary EEG systems can be integrated into so-called EEG easy caps.^31^ The latter option is also used in studies investigating typically developing infants.^32^ Here, we decided to use a neoprene cap that is thicker than a standard EasyCap. This was done to better hold the weight of a larger number of sensors when babies would lie down to sleep and minimize optical decouplings in such position. A recent PhD project combined a fiber-based broadband NIRS system with EEG into a neoprene cap for studies in awake infants and demonstrated good data quality.^34^

In summary, the choice of which systems to combine for integrated fNIRS-EEG depends on available systems and infrastructure for custom sensors. Most infant work incorporates separate electrodes and optodes into an existing cap. Of note in this manuscript, we refer to the stationary, non-mobile NIRS system as non-wearable and to the mobile fNIRS system as wearable.

#### 2.1.2 Phase 1: non-wearable NIRS system

**Fig. 1:**
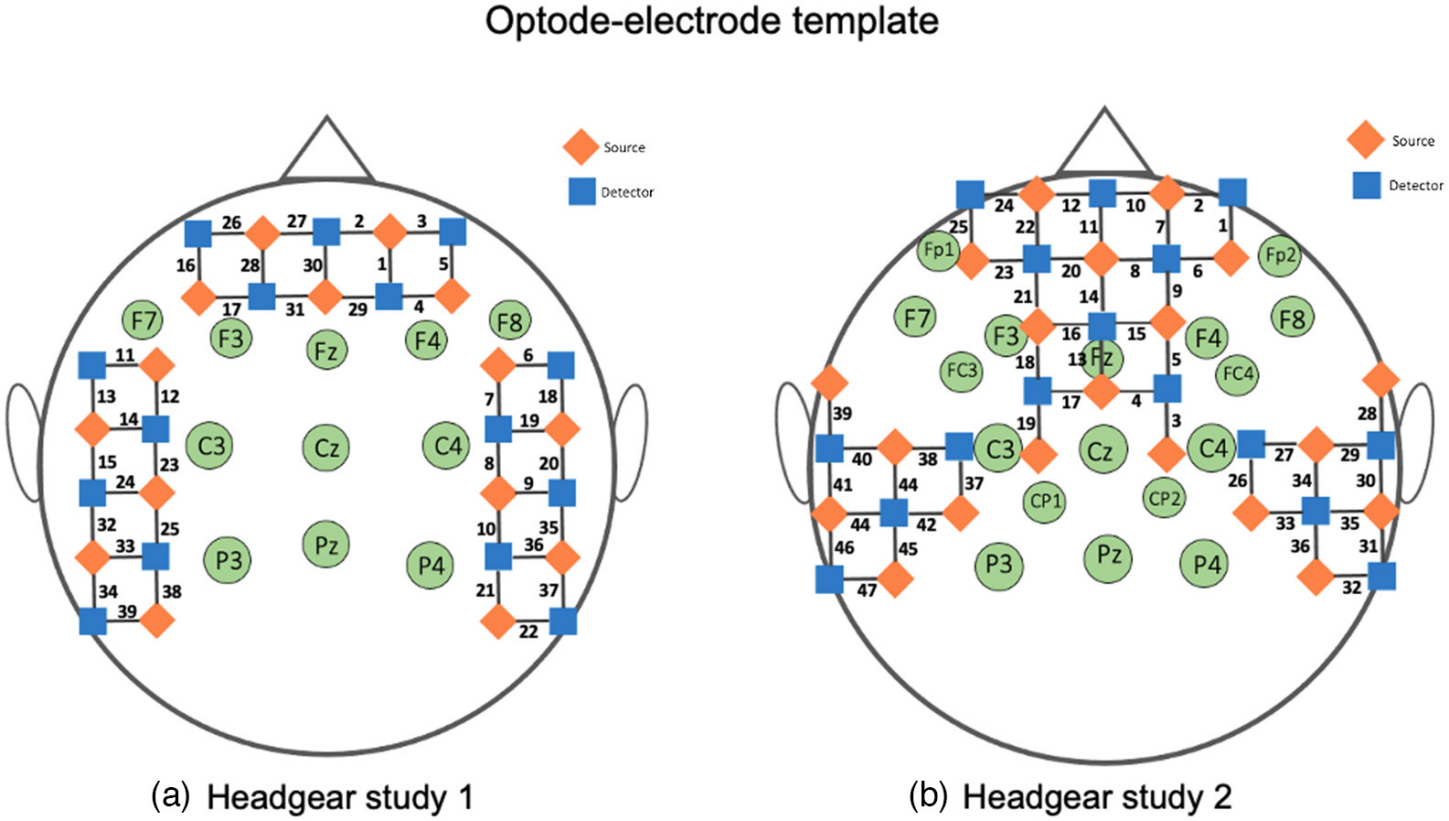
Optode-electrode template for (a) headgear study 1 and (b) headgear study 2.

In the present study, we initially modified neoprene caps used for wearable EEG testing (see Neuroelectrics, ES) to include optodes with the aim of preserving source–detector separation and integrating optodes with electrodes while ensuring cap robustness. The neoprene fabric is breathable and allows for extended wear time while addressing challenge 2. To enable the placement of EEG electrodes alongside the optodes, electrodes were placed on the midline with optodes distributed across the frontal and temporal lobes (1) to maximize coverage while leaving the back of the head free so that infants could be lying on their back and not be uncomfortable; (2) to be able to measure sleep EEG characteristics that are essential for sleep stage scoring, such as sleep spindles, which preferentially occur along the midline;^40^^,^^53^ and (3) to enable comparison with prior studies on fNIRS-MRI co-registration in infants of a similar age range and using the same fNIRS system and array for matching known underlying anatomical regions.^43^ See Fig. 1(a) for the optodes-electrode design. This design allowed for sufficient separation between the two systems to minimize the chances of gel leakage, also addressing challenge 1.

**Fig. 2:**
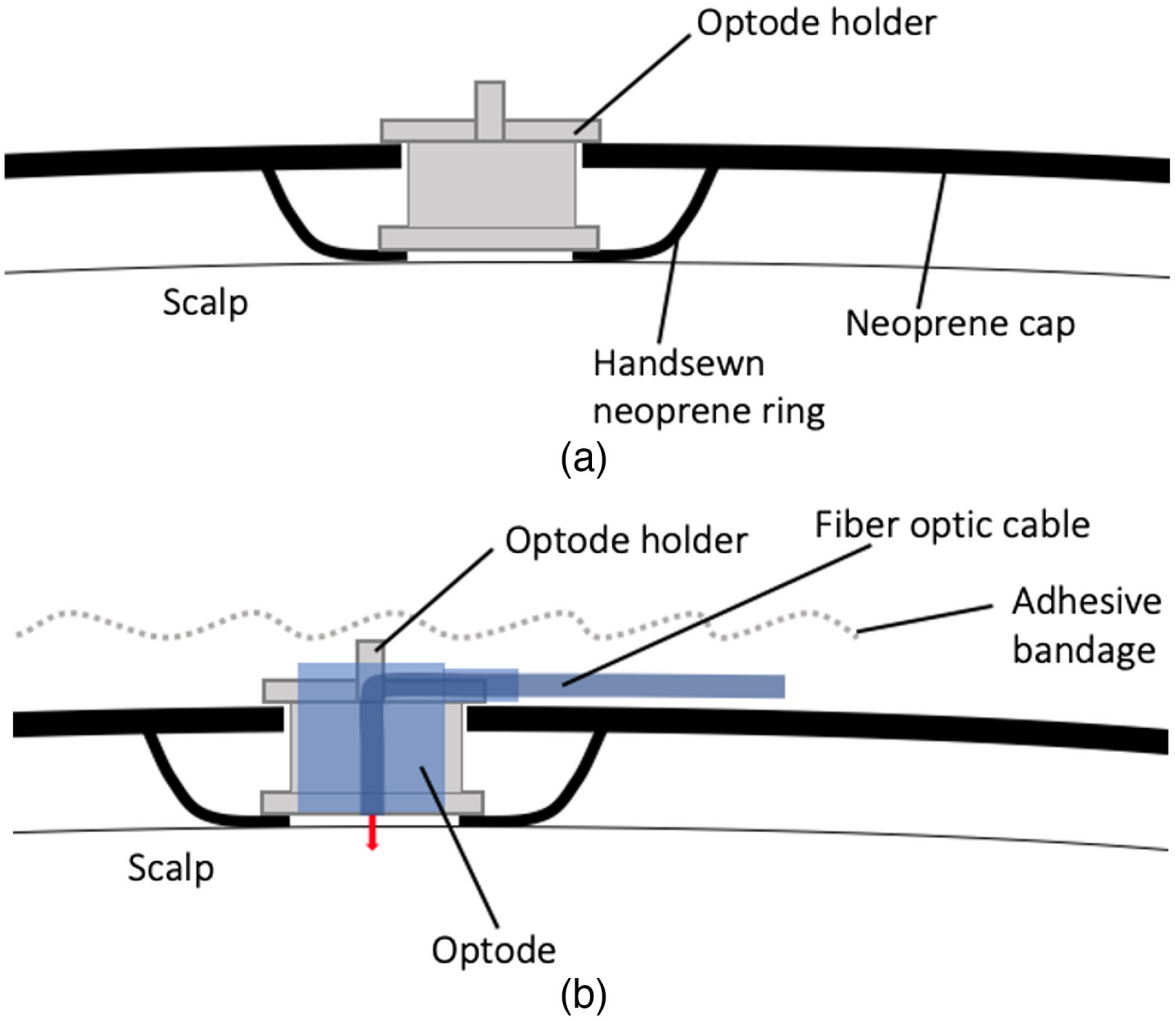
(a) Illustration of the optodes holder sitting in the hand-sewn neoprene ring. (b) Illustration of the optode holder and the optode sitting in the hand-sewn neoprene ring.

A 3-D printed custom optode holder^34^ was designed to have the same dimensions as the electrodes and was used to fit the optodes seamlessly into the neoprene cap. See Fig. 2(a) for an illustration. To address challenge 2, neoprene cloth rings were sewn to the inside of the headgear where optodes were sitting. Specifically, the plastic ring of the optode holder was covered by an additional layer of cloth while allowing the detector/source to make good contact with the infant scalp. The aim was to reduce the pressure of optodes onto the skin while maximizing the data quality. See Fig. 2(b) for an illustration. An adhesive bandage was wrapped around the entire headgear after placement to avoid infants pulling on the cables or getting caught in the optode holders and to further maximize optical coupling with the head.

#### 2.1.3 Phase 2: wearable NIRS system

**Fig. 3:**
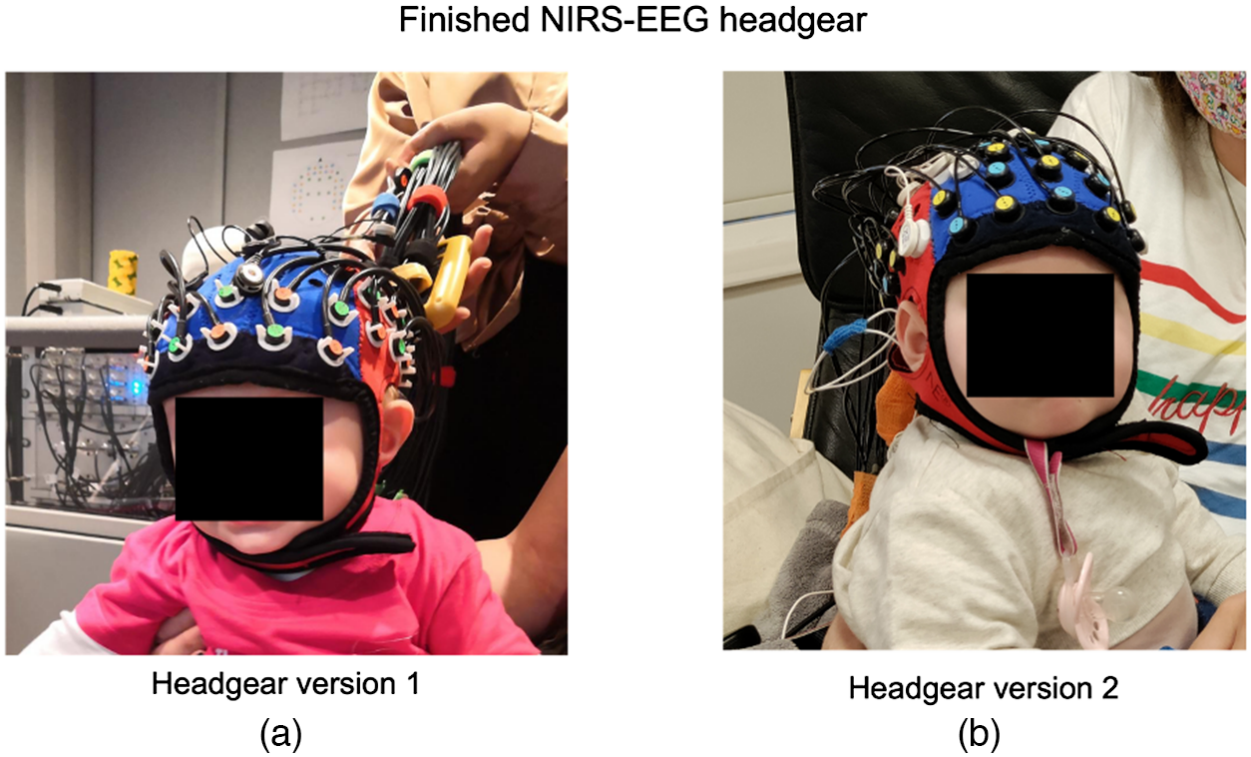
Finished headgear for (a) version 1 and (b) version 2.

As discussed further below, the use of the non-wearable system showed some caveats, e.g., infants not napping in the lab. In an evaluation of the protocol and system, we conjectured the problems to be rooted in the physicality of the heavy, unwieldy non-wearable NIRS system with the long, heavy fiber optic cables that made navigating and moving the participant more difficult and reduced the comfort of the infant and parent. Therefore, we recently tested whether the use of a wearable NIRS system would improve the participant’s comfort and the experimental setup. With a wearable EEG system already in place, the aim was to have a fully wearable NIRS-EEG headgear. Due to the availability of a larger number of channels, further optodes were added into the cap and more electrodes were fitted to take advantage of the reduced weight of the system. See Fig. 1(b) for an illustration. These changes resulted in a fully mobile, light weight-adaption of headgear version 1 with improved coverage of the prefrontal cortex and more electrodes. These changes resulted in a fully mobile, light weight-adaption of headgear version 1 with improved coverage of the prefrontal cortex and more electrodes. In particular, headgear version 2 provides 12 additional fNIRS channels (25 in total versus 13 channels of headgear version 1) and 4 additional EEG electrodes (9 in total versus 5 electrodes of headgear version 1) over the prefrontal areas specifically. In the result section, we describe data quality for both headgear designs in detail (Fig. 3).

### 2.2 Participants

N=49 infants [age range: 5 to 9 months (161 to 253 days), mean age: 204 days, SD = 31 days, and 22 = female] took part in the study at Birkbeck Babylab, London, United Kingdom. N=26 (mean age: 190 days, SD = 23 days, 11 = female, and 10 infants slept) were tested using the non-wearable version of the NIRS-EEG headgear with 13-channel EEG (ENOBIO, Neuroelectrics) and 39-channel NIRS (NTS, Gowerlabs) in phase 1. N=23 infants (mean age: 217 days, SD = 32 days, 11 female, and 21 infants slept) were tested with the wireless version of the headgear with 20-channel EEG and 47-channel NIRS (BabyBrite, Artinis Medical Systems BV) in phase 2.

### 2.3 Experimental Procedure for a NIRS-EEG Infant Sleep Study

**Fig. 4:**
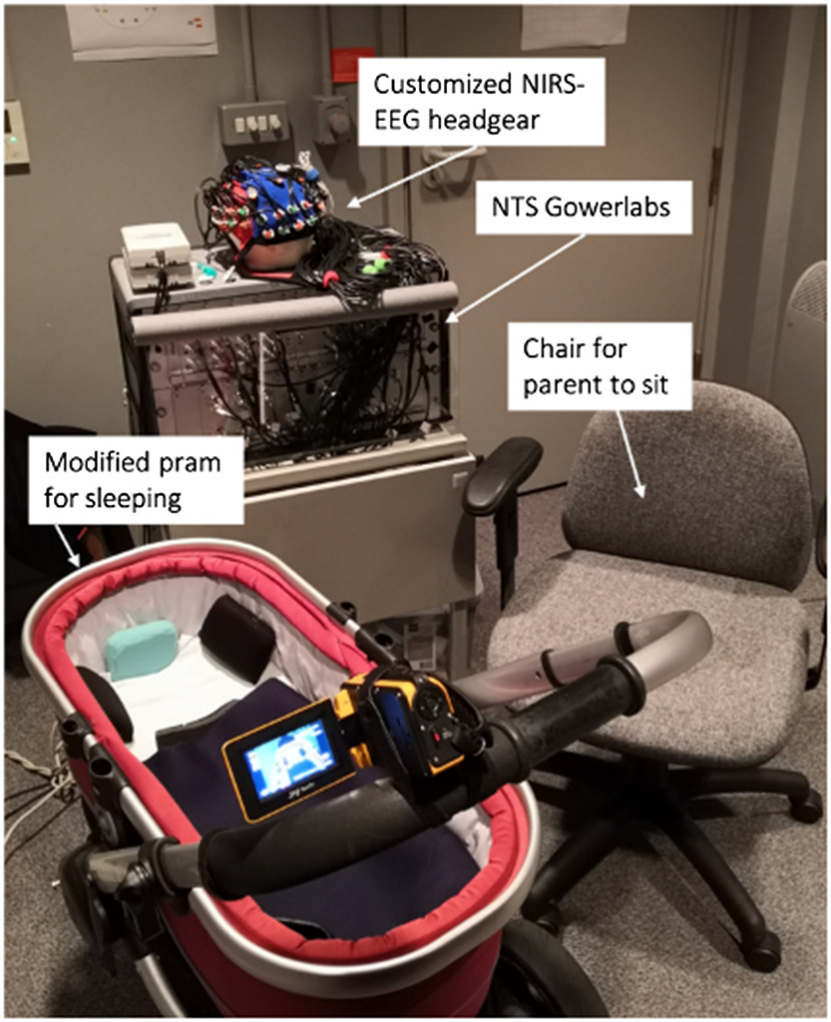
Sleep environment for NIRS-EEG infant sleep study.

The described protocol is part of a larger study that also included a home-based sleep assessment (actigraphy, sleep diary, and habitual sleep questionnaires, such as the Brief Infant Sleep Questionnaire (BISQ^54^) and eye-tracking, which are not further described here. An initial phone screening assessed the infants’ habitual sleeping patterns, in particular potential nap times and routines surrounding napping. The experimental setup of both studies was the same and was crucial to addressing challenge 3. The lab visit was scheduled approximately 45 min before the infant’s regular nap time and was set to take between 1 and 3 hrs depending on whether the infant slept or not. After welcoming the baby and the caregiver, they were accompanied to the room where the stroller and the PSG equipment (including customized NIRS-EEG headgear) were located (see Fig. 4). Regular pre-nap routines were replicated in the lab environment as closely as possible, e.g., caregivers were asked to bring their infants’ blanket/sleeping bag, familiar toys or plush animals, and familiar music/white noise. After putting our PSG equipment [NIRS, EEG, respiratory belt, and electromyography (EMG) electrode] on the infant, he/she was put into the stroller. If the infant usually slept in the caregiver’s arms, infants were held for the duration of their nap by their parent. Thereafter the researcher waited until the infants woke up again, recording the duration of the sleep. If it became apparent that an infant would not sleep, usually the decision was to wait 30 min and proceed with the remaining study protocol. We based the decision to wait 30 min in case of no sleep on parents’ feedback in the piloting process of the current study. Usually after 30 min of attempting to get the child to sleep without success, parents indicated that it would be unlikely for them to get the child to fall asleep. Further, sleep onset latency (i.e., the time it takes them to fall asleep) in infants this age is considered especially long if its longer than 20 min.^55^ See Supplementary Material for a detailed step-by-step guide on how to successfully conduct infant sleep studies using NIRS-EEG.

### 2.4 Technical Equipment

#### 2.4.1 EEG system

We used the 20-channel wearable Enobio System (Neuroelectrics, BCN, ES) with gel-based electrodes embedded in a soft neoprene cap and a Bluetooth transmitter with a sampling frequency of 500 Hz. We combined 13 EEG channels with the non-wearable NIRS system (phase 1) and all 20 EEG channels with the wearable NIRS system (phase 2).

#### 2.4.2 Non-wearable NIRS system

The non-wearable NIRS system was a 39-channel continuous wave NIRS system^56^ (NTS, Gowerlabs, United Kingdom) using wavelengths 780 and 850 nm. The acquisition sampling rate was 10 Hz. Optodes were arranged following the configuration, as shown in Fig. 1(a). The source–detector separation was set at 2 cm. The weight of the NTS fibers with the EEG system is ∼1000  g.

#### 2.4.3 Wearable NIRS system

Our wearable NIRS system combined two BabyBrite systems (Artinis Medical Systems BV, Netherlands) with 22 channels each using wavelengths 760 and 850 nm. The acquisition sampling rate was 25 Hz. Optodes were arranged in the configuration as shown in Fig. 1(b). The source–detector separation was set at 2 cm. The wearable NIRS system used lab streaming layer (LSL) for data acquisition. The weight of both BabyBrite systems in combination with the EEG system is ∼400  g.

### 2.5 Protocol for Evaluating fNIRS Data Quality

To evaluate the feasibility of the proposed EEG-fNIRS headgears, we assessed the quality of the data that can be recorded with the developed solutions. fNIRS data quality checks and pre-processing were performed using Homer2 and custom-written scripts. Data were first segmented to only include sleep data based on the experimental protocol logs of timings of when infants were asleep.

**Fig. 5:**
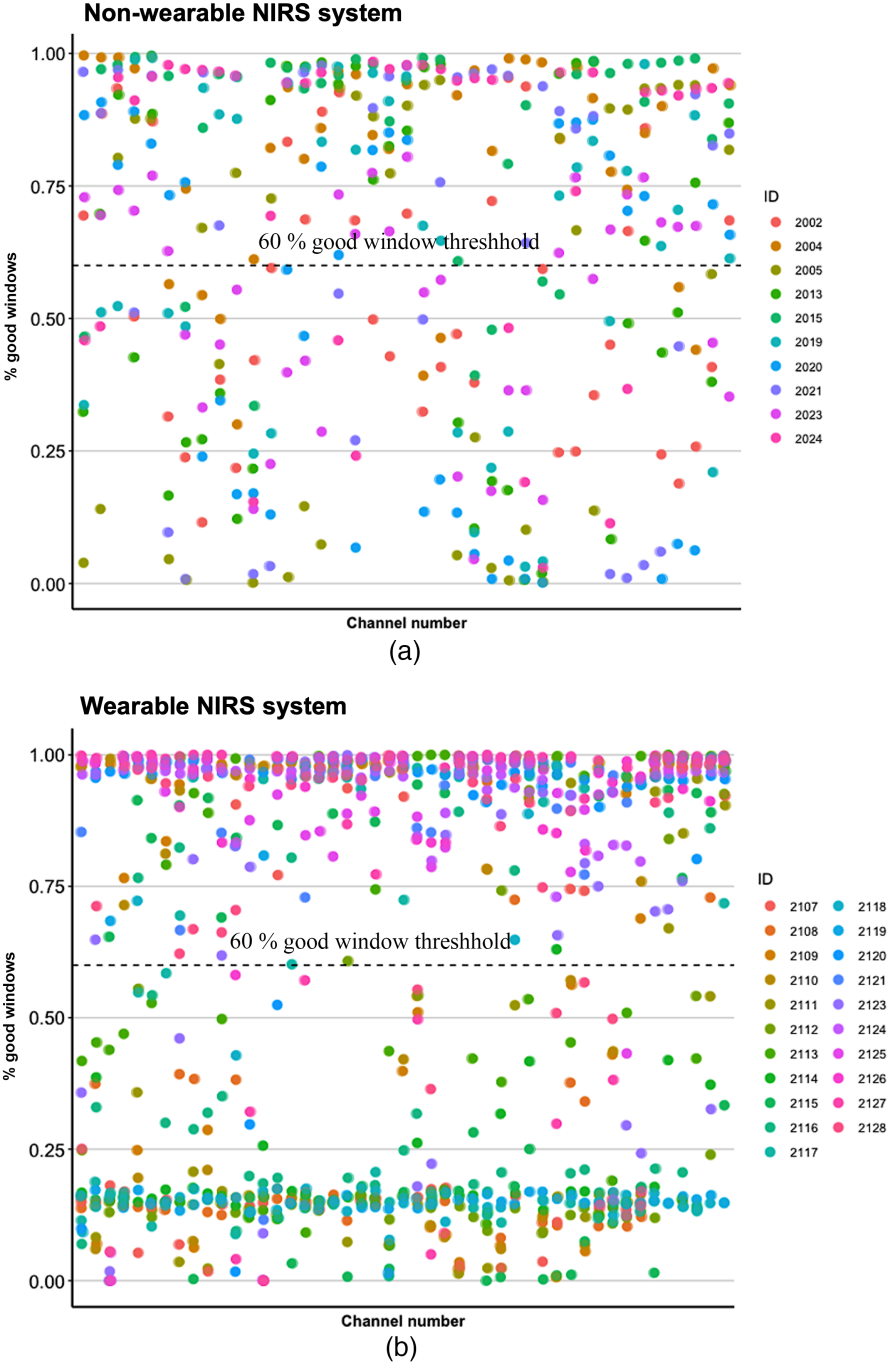
Percentage of good windows for (a) non-wearable NIRS system and (b) wearable NIRS system.

In agreement with the guidelines provided by Germignani and Gervain, raw fNIRS intensity signals were first converted into changes in optical density and then pre-processed using the Homer2 software package.^57^ The pre-processing pipeline is summarized in Fig. 5. In particular, motion artifacts were corrected by combining the spline-based and the wavelet-based algorithms to maximize data retention,^58^ as recommended for infants’ fNIRS data,^59^ and a band-pass filter (0.01 to 0.5 Hz) was used to minimize high frequency and very low frequency physiological noise. Pre-processed optical density signals were then converted into changes in concentration of HbO2 and HbR.

The fNIRS data quality was assessed using a novel tool called QT-NIRS “(Quality Testing of NIRS;” an extension of the software package PHOEBE;^60^ https://github.com/lpollonini/qt-nirs), developed by Luca Pollonini and his lab at the University of Houston.^61^^,^^62^ QT-NIRS excludes channels based on insufficient correlation between the two wavelengths and the absence of a clear heart rate under the assumption that the heart rate component is visible in the fNIRS signals when there is a good optical coupling between the optodes and the head. The correlation and its strength are expressed by two indices: the scalp contact index (SCI) and the peak spectral power (PSP). Recently, Bulgarelli et al. showed that this approach yielded significantly better signal-to-noise ratio than previously used methods.^63^

We ran the QT-NIRS pipeline on the sleep segments for data acquired by both the non-wearable and the wearable versions of the headgear. The SCI and PSP indices were calculated within not overlapping time windows (3 s in this case), and time windows were flagged as high quality if both SCI and PSP exceeded a threshold (i.e., $\theta_{\mathrm{SCI}}$ and $\theta_{\mathrm{PSP}}$, respectively). The fNIRS channel was then excluded if it did not reach the scan quality threshold $\theta_{\mathrm{SQ}}$, i.e., 60% percentage of time windows needed to be flagged as high quality. Here, we used $\theta_{\mathrm{SCI}}=0.6$, $\theta_{\mathrm{PSP}}=0.1$, and $\theta_{\mathrm{SQ}}=60\%$.

Our criteria for assessing the fNIRS data quality were as follows.

1. Percentage of good time windows based on QT-NIRS identification, i.e., the number of time windows flagged as high quality and hence above the $\theta_{\mathrm{SCI}}$ and $\theta_{\mathrm{PSP}}$ thresholds.
2. Number of included channels.
3. Percentage of time periods of the whole recording corrupted by motion. We marked the number of samples marked as motion by Homer2 in the fNIRS data, i.e., the time periods corrupted by motion. The number of motion periods is normalized by the total duration of the signal.
4. Duration of nap (the duration of the nap was calculated based on the observation of the infant’s behavior, i.e., nap onset when the babies closed their eyes and nap offset when they opened their eyes and started moving).
5. Percentage of overall valid EEG data that can be used to classify sleep stages after basic filtering (see Sec. 2.6.).

In addition, we also report the location of sleep (cot/pram versus parent’s arms) and aim to understand whether the quality metrics of either of the two headgears give bias in the data based on habitual sleep patterns (toward good or poor habitual sleepers) by looking at the habitual sleep data that we have available (BISQ). Figure S1 in the Supplementary Material shows two examples of how habitual sleep data relate to the two headgears.

Finally, raw fNIRS intensity signals from the included channels were converted into changes in optical density and corrected for motion artifacts^59^ and physiological noise.

### 2.6 Identification of Valid EEG Data

EEG data were filtered using EEGLAB version 19.1. Data were bandpass filtered at 0.4 to 25 Hz. EEG data were then assessed as to whether it was possible to classify sleep stages, a process which is done by manual sleep stage scoring by a trained expert sleep scorer, as is gold standard practice in sleep research.^53^^,^^64^ In sleep staging, data are viewed in 30 s epochs, and the expert scorer decides on the sleep stage. They are guiding by a set of standardized rules outlined in the American Manual for Sleep Stage Scoring,^53^^,^^64^ and it involves looking for characteristics of specific sleep stages (e.g., presence of sleep spindles for stage 2 sleep). Examples of valid EEG data of different sleep stages as well as examples of EEG data of too poor quality for sleep staging to be possible can be found in the Supplementary Material. We consider EEG datasets to be invalid if they contained too much noise in the data to reliably and consistently identify sleep stages across most 30 s epochs. Figure 7 below and Fig. S4 and Sec. 4 in the Supplementary Material show an example of one subject (from study phase 2) to show how EEG sleep data could be linked to fNIRS sleep data in future studies. Figure 7 also includes data samples of fNIRS as well as EEG data for a single subject.

## 3 Results

### 3.1 Percentage of Good Time Windows Based on QT-NIRS

#### 3.1.1 Non-wearable system

The average good window percentage across all channels was 64.2%. (SD = 13.1%). Figure 5(a) shows an illustration of the individual good window percentages per channel in the non-wearable system.

#### 3.1.2 Wearable system

The average good window percentage was 57.7% (SD = 26.5%). Figure 5(b) shows an illustration of the individual good window percentages per channel in the wearable system.

**Fig. 6:**
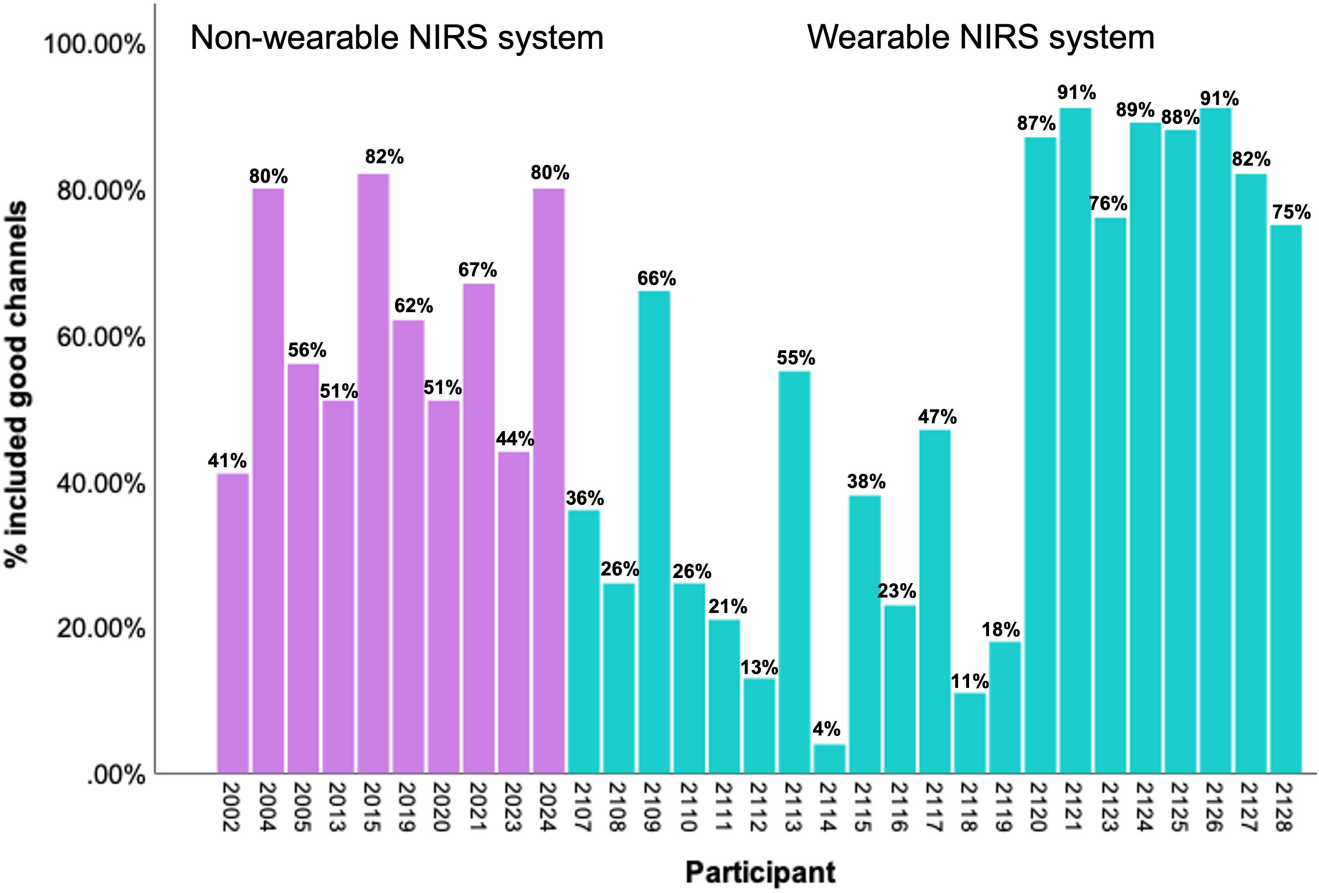
Percentage of included good channels for each participant. Note: parameters: $\theta_{\mathrm{SCI}}=0.6$; band-pass filter [1.5 3.5] Hz; $\theta_{\mathrm{PSP}}=0.1$; and $\theta_{\mathrm{SQ}}=60\%$. Window length = 3 s.

Overall, the average good window percentage classified by QT-NIRS did not significantly differ between systems [equal variances assumed: t(29)=0.724, p=0.47 | equal variances not assumed: t(29) = 0.906, p=0.373, Cohen’s d=0.28]. See Fig. 6 for an illustration. Figure 6 suggests that there is a disparity in good time windows in the wearable system with some channels being either poor or good continuously rather than alternating between good and poor quality. This may indicate that less optical decoupling occurs with the fully wearable headgear.

### 3.2 Number of Included Channels

**Fig. 7:**
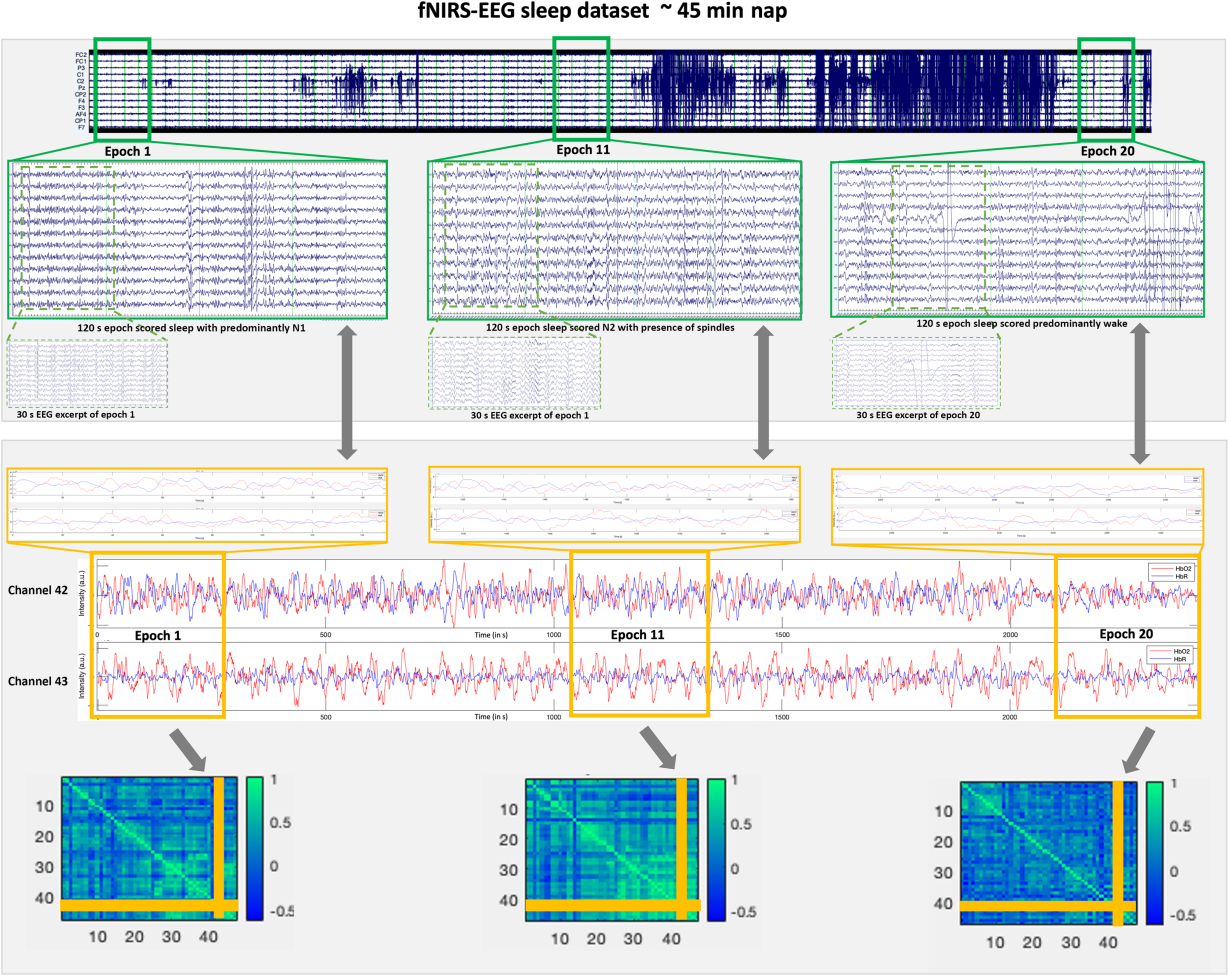
Example of single subject nap data for EEG data and fNIRS data. For EEG, all good quality channels are displayed. For fNIRS exemplary channels 42 and 43 are shown. Note: s = seconds.

Figure 7 shows the included channels based on QT-NIRS in study phase 1 and phase 2. Study phase 1 (mean = 61.4%, SD = 15.32) provides more included channels based on QT-NIRS than study phase 2 (mean = 50.6%, SD = 31.14), though this difference is not statistically significant [t(29) = 1.03, p=0.311, Cohen’s d=0.4].

### 3.3 Percentage of Motion-Corrupted Time Periods

For the non-wearable NIRS system, the percentage of motion corrupted time periods across all good channels was 1.9% (SD = 1.3%). For the wearable NIRS system, the percentage of motion corrupted time periods across all good channels was 3.4% (SD = 2.3%). The percentage of motion corrupted time periods was not significantly different between study phase 1 and study phase 2 (t(30)=−1.96; p=0.06).

### 3.4 Nap Duration, % Infants Asleep, and Habitual Sleep Duration

In general, infants with poor parent-reported sleep did not nap with the headgear on if they came into the lab, and parents of infants with poor sleep did not sign up to the study in the first place.

#### 3.4.1 Non-wearable system

Nap durations for the non-wearable system ranged from 23 to 62 min (mean = 36.9 min; SD: 12.9 min). 42% of infants tested slept with the headgear on. Most infants in study phase 1 napped in the pram. Habitual sleep data information was available from N=10 infants. Table S1 in the Supplementary Material shows descriptive statistics for habitual sleep.

#### 3.4.2 Wearable system

Nap durations for the wearable system ranged from 17 to 105 min (mean = 56.11 min; SD: 24.86 min). 90.9% of infants tested slept with the headgear on. The majority of study phase 2 infants napped in the parents’ arms with two infants napping in a cot and one in a pram.

Habitual sleep data information was available from N=15 infants. Table S1 in the Supplementary Material shows descriptive statistics for habitual sleep.

Overall, infants slept significantly longer with the wearable system than the non-wearable system (t(29)=−2.47, p=.02).

### 3.5 EEG Data

For information on how EEG data were assessed, see Sec. 2.6. For an illustration on how to link EEG data to fNIRS data in next steps, see Sec. 4 in the Supplementary Material and Fig. 7 below. Figure 7 also includes data samples of fNIRS as well as EEG data for a single subject.

#### 3.5.1 Non-wearable system

45% of EEG data could be used for EEG sleep stage classification after basic filtering.

#### 3.5.2 Wearable system

100% of EEG data could be used for EEG sleep stage classification after basic filtering.

## 4 Discussion

We developed a customized fNIRS-EEG headgear and tested the feasibility of using this system in 5- to 9-month-old sleeping infants. Prior research has used such fNIRS-EEG systems for neonates in hospital settings and have shown that connectivity patterns fluctuate across the duration of sleep in newborns and in adults and that those fluctuations might reveal more information about and map onto underlying sleep stages. Moreover, the combination of fNIRS and EEG could reveal important information about how investigating connectivity during sleep could provide insight into the relationship between sleep and development. Before functional connectivity can be investigated in relation to sleep markers, there was need for a feasibility study and method development of fNIRS-EEG for infant sleep research.

To this goal, we tested a non-wearable and a fully wearable version of the headgear and evaluated the quality of the fNIRS and EEG data that can be recorded using both on sleeping infants. It was possible to use both versions of the customized NIRS-EEG headgear (i.e., both the non-wearable and the wearable NIRS systems) for sleep recording in 5- to 9-month-old infants. Below we evaluate the results of each headgear version separately.

### 4.1 Evaluation of Non-Wearable Headgear Performance

The rate of infants who slept in study phase 1 was around 40%, something that is acceptable for infant sleep research based on prior research. Infants who slept with the headgear on usually had (parent-reported) no more trouble sleeping than normally and seem to tolerate it as well as similar caps are tolerated in infant populations. Some infants who slept for a longer time showed pressure marks, which faded within half an hour after removal of the cap. Overall the instructions for parents to bring their child’s familiar sleep environment worked well. The study was time-consuming (5 hrs/participant) and always required two researchers. Moreover, the heavy fiber bundles of the non-wearable system used in this study meant that parents could not just pick up their infant when they needed to be soothed during sleep, resulting in some infants sleeping shorter than they typically would. The fiber-bundles made feeding infants to sleep a challenge and did not allow for parents to hold their infants easily or move around with the infants. Even with the use of an arm to hold the bundles, the weight of the fibers still represented a limitation. Of note, even non-wearable systems with less heavy fiber bundles than the system used in this study would impede moving around with the infant as part of the bed-time routine or, for example, putting infants to sleep in the pram and moving the pram around.

Second, the data quality from the study was mixed. The NIRS data quality was better than the EEG data quality. NIRS data in the infants that slept (N=10) was good; however, only 45% of those infants had EEG data that could be used for sleep stage scoring. This might have been because EEG mastoid reference placement was harder with the heavy cap, or as all EEG electrodes were placed along the midline, the cap fit might have been looser toward the top of the head with the heavy optical fibers pulling up the electrodes and reducing scalp contact of the electrodes. fNIRS data showed some poor-quality channels, though typical for fNIRS infant studies. Infants were lying on the side in the pram, which could have reduced data quality on one side particularly. In addition, we noticed that optical decouplings were more frequent with this version of the headgear. This was likely because the long and heavy headgear fibers pulled substantially when the infant moved during sleep, reducing scalp contact.

### 4.2 Wearable NIRS-EEG for Infant SLEEP Research

The rate of infants who slept with the wearable NIRS-EEG was >90%, which was higher than for the fiber-based NIRS. More infants slept in the study, and sleep duration was also longer. However, data quality was much more variable than with the fiber-based system, and some data sets showed poorer quality. However, the difference in data quality between the two systems was not statistically significant. The drop in data quality for some infants may be due to the fact that some parents held their infants while sleeping in this study, potentially introducing additional noise to the data. Thus, although more infants slept, future research needs to ensure continuous good data quality. The wearable headgear allowed for parents to interact more freely with their child, allowing for feeding or position changes. In addition, we found that the wearable solution gives the opportunity to measure in a situation that is more similar to a real world situation, as might be seen in the scatterplot of the fNIRS measures with behavioral sleep measures. (See Fig. S1 in the Supplementary Material, a scatterplot that illustrates the relationship between the amount of good quality time windows of fNIRS data might differ depending on habitual sleep characteristics in the non-wearable system but not in the wearable system. However potentially no associations were significant, likely due to the low N. We interpret this to mean that, using the wearable system, infants are more likely to sleep as they would at home.) The use of this technology may thus hold potential to increase the representativeness of infants included in the study. Our analysis of motion-corrupted time windows showed no statistically significant difference of motion artifact presence between the non-wearable and wearable systems.

The non-wearable NIRS system of phase 1 might provide slightly better data quality being a lab-based instrument incorporating more sophisticated optical components, such as laser diodes and avalanche photodiodes, whereas the wearable NIRS system features light-emitting diodes (LEDs). Laser diodes provide higher spectral resolution, higher sensitivity, and penetration depth,^65^ with narrower emission spectra than LEDs (e.g., 1 nm versus 30 to 35 nm^65^^,^^66^), hence potentially providing better fNIRS data quality. However, lasers often require cooling systems to reduce heating of the components and optical fibers to guide light to the head; being highly collimated, they may be less safe to the human eyes. By contrast, the light emitted by LEDs is uncollimated and incoherent and can thus provide measurements at higher intensity levels; they are also cheaper, compact, able to be battery powered, and safer for use on infants. Therefore, wireless fNIRS technologies implementing LEDs represent a good compromise in terms of data quality and safe and mobile recordings.^66^^,^^67^

### 4.3 Potential Biases in Infant Sleep Research

As alluded to above, testing sessions using the customized NIRS-EEG headgear carried a number of (potential) biases that warrant discussion. For infants sleeping in the pram and infants that were parent-reported easy sleepers/good sleepers, the non-wearable NIRS worked very well. For poorer sleepers (indicated by the parent during a pre-study phone call), the wearable system worked better as it allowed parents to interact more freely and naturalistically with their child (including feeding or rocking to sleep). Interestingly, when the preliminary habitual sleep questionnaire data were examined from the present study, there were no differences in habitual sleep fragmentation or sleep duration (day or night) markers between infants who napped in the lab versus infants who did not nap in the lab. We interpret these results as evidence that it is possible to get representative data across both habitually good and poor sleepers. However, parents who reported that their child would likely not sleep in the lab were generally accurate. Future work will include (1) using a larger sample and (2) investigating whether sleepers and non-sleepers differed in habitual sleep using actigraphy data rather than sleep questionnaire data (e.g., BISQ), to account for the potential cross-method differences between actigraphy and sleep questionnaire data (e.g., BISQ). The issue of breastfeeding infants to sleep is an important bias to consider, too. These considerations become especially important when thinking about expanding the testing age range further beyond the first year of life. Infants who were breast-fed fell asleep more readily than infants who were not breastfed. This could introduce a systematic bias into the sample. Future work should take a closer look at the role of biases that might affect the data collected.

### 4.4 Limitations and Future Use for NIRS-EEG Headgear in Infant Sleep Research

Overall, our study demonstrated the feasibility of using a non-wearable as well as a wearable fNIRS-EEG headgear in sleep research. The fNIRS data quality was sufficient to conduct further analyses, and a large percentage of the EEG data collected allowed for sleep stage classification. Future studies will evaluate the extent to which this approach sheds light on the association between sleep and development.

We acknowledge that this study used NIRS systems and an EEG system available to the authors at the time of the study, but the steps taken to design a customized headgear comfortable enough for sleeping infants can be generalized to different types of wearable NIRS and EEG systems. Based on the detailed step-by-step guide in the Supplementary Material, we think this headgear and study can be recreated with other devices. Previous studies have shown that NIRS-EEG can be acquired from sleeping newborns using DOT/fNIRS systems, e.g., Ref. 68. We think these systems have great potential to provide unique information, such as tomographic maps of naps or overnight sleep in studies of older sleeping infants, too. However, several issues will have to be addressed before the systems can be transitioned into use with older infants for the purpose of studying sleep. These include, for example, wearer comfort during sleep, which is a bigger issue in older infants due to reduced sleep pressure, and higher awareness to environmental stimuli. Integration with EEG is also potentially more difficult with high density fNIRS systems as there is less space to integrate the EEG electrodes in given locations.

The infants in this study were ages 5 to 9 months old, but internal testing shows that headgear version 2 might also be used to study sleep in toddlers. Future work should focus on testing our setup in sleeping pre-school children and toddlers.

We also found that (1) infants with poor parent-reported sleep did not nap with the headgear on if they came into the lab and (2) parents of infants with poor sleep did not sign up to the study in the first place. As a first feasibility study, this might not be an immediate issue; though, for future studies that aim at disentangling the association between sleep and development, it will present further challenges.

Along those lines is the advantage that the headgear version 2 (wearable headgear) allows for in-home measurements. Infants with poor sleep quality and older infants/toddlers might sleep better when sleep is measured in the comfort of their homes. Finally, in-home measurements also will enable researchers to reach a diverse range of socioeconomic backgrounds and different cultures.

### 4.5 Conclusion

In this study, we demonstrated the usability of an integrated NIRS-EEG headgear with both a fiber-based and wearable NIRS system. We outlined an efficient experimental protocol to conduct NIRS-EEG infant sleep studies to achieve good data quality and proved that it can be used for sleep recording in infants 5- to 9-month-old. The fiber-based NIRS system of study 1 might provide better data quality as a lab-based instrument due to its laser diodes and avalanche photodiodes, though this difference was not statistically significant. Meanwhile, the wearable NIRS system features LEDs due to its need for battery powered miniaturization that does not allow for laser diodes, potentially providing worse data quality. However, in pediatric sleep studies, the priority is an infant’s comfort and allowing a family to freely interact around nap time, which in the long run provides data from every infant not only from good sleepers. This is crucially for ecologically valid pediatric sleep studies. Furthermore, the use of this headgear can be extended to other populations for sleep research purposes, such as toddlers, and can be used for in-home or field testing. Thus, we think that the wearable NIRS-EEG headgear represents a good compromise between data quality, opportunities of applications (home visits and poor sleepers), and experiment success (infants’ comfort, longer sleep duration, and increased opportunities for caregivers to interact with their child).

## Code, Data, and Materials Availability

Analysis code and data used in this study will be made available upon request to the corresponding author. Materials transfer agreements may have to be signed.
