# Supplementary material for: Developing customized NIRS-EEG for infant sleep research: methodological considerations
Source: Neurophotonics. 2023 Sep 25;10(3):035010. doi: 10.1117/1.NPh.10.3.035010 (PMC10519625; doi:10.1117/1.NPh.10.3.035010)
Supplement: Supplementary file 1 [file NPh_010_035010_SD001.pdf]

Supplementary material

Developing customized NIRS-EEG for infant sleep research: methodological considerations

Louisa K. Gossé[a*], Paola Pinti[a], Frank Wiesemann[b], Clare E. Elwell[c], Emily J.H. Jones[a]

SM Section 1: Conducting an Infant sleep study using NIRS-EEG - standard operating procedure

Section SM 1.1 Headgear construction

Materials:

- neoprene caps (sizes 42cm, 45cm for ages 5-9 months)
- Neoprene fabric 2mm thick (black, dark blue) or similar sturdy, breathable material
- sewing materials
- hole puncher
- measure tape/digital calliper
- optode holders (custom 3-D printed or provided by fNIRS company)

Step 1: Decide on the optodes and electrodes configurations to use.

a. Consider the importance of measuring midline EEG during sleep for the purpose of collecting information on sleep spindle characteristics to be able to score different sleep stages.
b. Maximize fNIRS coverage by spreading optodes across the brain.
c. Ensure adequate distance between fNIRS optodes and EEG electrodes if gel-based electrodes are used.
d. Decide on source detector separation based on subject age.
e. Consider how and where the majority of infants will nap (e.g., unless there is a good reason to measure the occipital cortex, avoid placing sensors at the back of the head to make infants more comfortable).
f. Where possible, replicate arrays that have been used in other fNIRS studies of similar age and system.

Step 2: Measure out the all optode locations and EEG locations on the neoprene cap when the cap is stretched on an age-matched head model, to account for the potential stretching of the material that may change source-detector separation slightly. Use the hole puncher to cut all the optode holes.

Step 3: Depending on the material of your optode holders, you may want to cover the optode holders edges with fabric material by sewing a ring on the inside of the neoprene cap (glueing will not work and will make the cap rigid, uncomfortable and less breathable). See below (and main manuscript) for illustration.

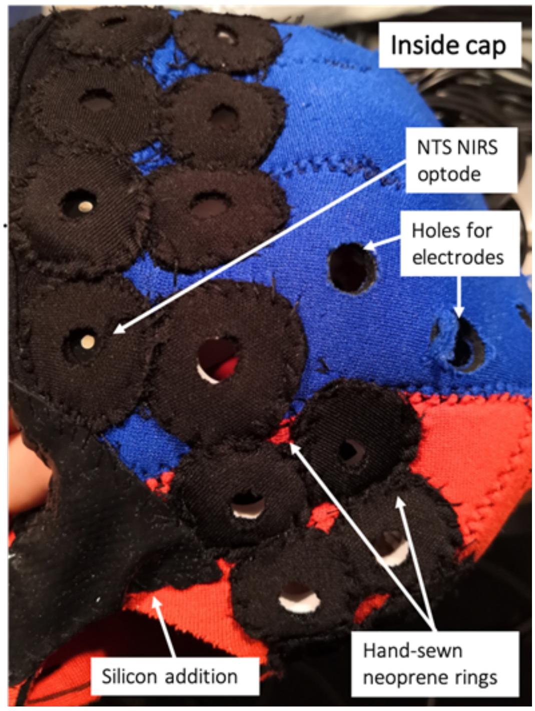

Step 4: Add in both the EEG and fNIRS, key is to separate both systems.
Step 5: Synchronizing EEG and fNIRS data streams

a. This can be achieved based on the hardware used and can be network-based or wired-based. For wired solutions, send a TTL trigger simultaneously to both the fNIRS and EEG acquisition units. For network solutions, use network protocols like LabStreaming Layer (https://github.com/sccn/labstreaminglayer) to send an event marker to both the EEG and fNIRS (this is the solution adopted in this study). This will enable the realignment of the fNIRS and EEG recordings with millisecond precision.

Step 5: Conduct interference tests
Step 6: Decide on additional EEG sensors (e.g., EOG, EMG,…) and reference placement (e.g., mastoids, …) as well as heart rate or respiration measurements.

Section SM 1.2 Conducting an infant sleep study

<u>**Step 1:** Before the study - Room set-up</u>

The room where the sleep study should be:

a. as unexciting and bland as possible
b. without natural light and with a light dimmer to be able to control light intensity
c. quiet and away from other (noisy) studies
d. should have a comfortable chair for parents to sit in or a sofa
e. cot for baby or even a bed that also accommodates parent

f. pram that is modified to be able to accommodate placement of the devices next to the baby or a bag where the devices can be placed to be hanging from the side
g. prepare sleep lullabies, light sleep toy, white noise, blankets for baby and parent, potentially a feeding pillow

<u>**Step 2:** Before the study: family & baby requirements</u>

Understanding family environment and sleep habits prior to scheduling a sleep study visit is key.

- ask the parent to measure the baby's head to prepare the cap with the right size in advance and save time during the testing session
- enquire about sleep clothing, habits such as breastfeeding and instruct parents to bring anything the baby is familiar with in terms of their sleep routine and habits. Try to replicate as closely as possible in the lab environment.
- Schedule the visit approximately 1 hour before sleep onset.
- Make sure parents understand that their baby might take a while to fall asleep or that they may not sleep at all. This will take pressure of the parent (and the researcher!).

<u>**Step-by-step lab visit**</u>

1. welcome family
2. keep things calm and quiet throughout the beginning of the visits
3. try to not provide very stimulating toys or TV unless absolutely necessary
4. make sure that the family has plenty of time to get settled into the room
5. always have 2 researchers there for capping the baby
6. ask parents for cues as to when baby might feel sleepy (signs could include e.g., increased fussiness, rubbing eyes, ears,…)
7. put baby in sleeping bag/pyjamas (if parents wish to do so)
8. give the opportunity for solid food and/or nappy change BEFORE capping
9. pre-gel the electrodes in the NIRS-EEG cap right before putting the cap on the baby
10. do the capping while baby on caregivers lap facing researcher 1, there will be one try only, due to EEG gel leaking onto scalp (bridging, worse fNIRS quality),
11. researcher 1 in front of baby and put cap on baby, aligning frontal optode array with anatomical markers (e.g., Nz) while researcher 2 is holding the EEG/fNIRS cables off the babies head. do not close the cap yet if you are using mastoid references.
12. place mastoid references behind the ear and attach the chin strap.
13. use coban wrap to cover the sensors and tighten loose caps by wrapping several times around babies head (see picture below).
14. breastfeeding/Milk if applicable AFTER capping
15. settle baby into initial sleeping position
16. consult with parents regarding light level and noise or if the pram should be moved to rock baby to sleep
17. put headgear on before feeding the baby but wait to calibrate sources and EEG system and to start recording until baby is in " final sleep position"
18. be patient. It may take babies up to 1 hour to fall asleep. Some babies cry when they are trying to fall asleep.
19. if applicable place other sensors such as EMG/EOG, when baby is asleep or drowsy and after breastfeeding.
20. Start recording as soon as the baby is in the final sleeping position.

21. Ask parent to report on when they think their baby has fallen asleep and monitor EEG signal for sleep onset.
22. Let baby sleep until they naturally wake up.
23. Report baby wake up time.
24. Stop data recording.
25. Take pictures of cap placement when baby has woken up.
26. Take off cap and wipe head clean of gel, make sure to gently remove the reference stickers using baby oil.

r = r.29,p = .29,0p = .40

Section 2: Habitual Sleep data

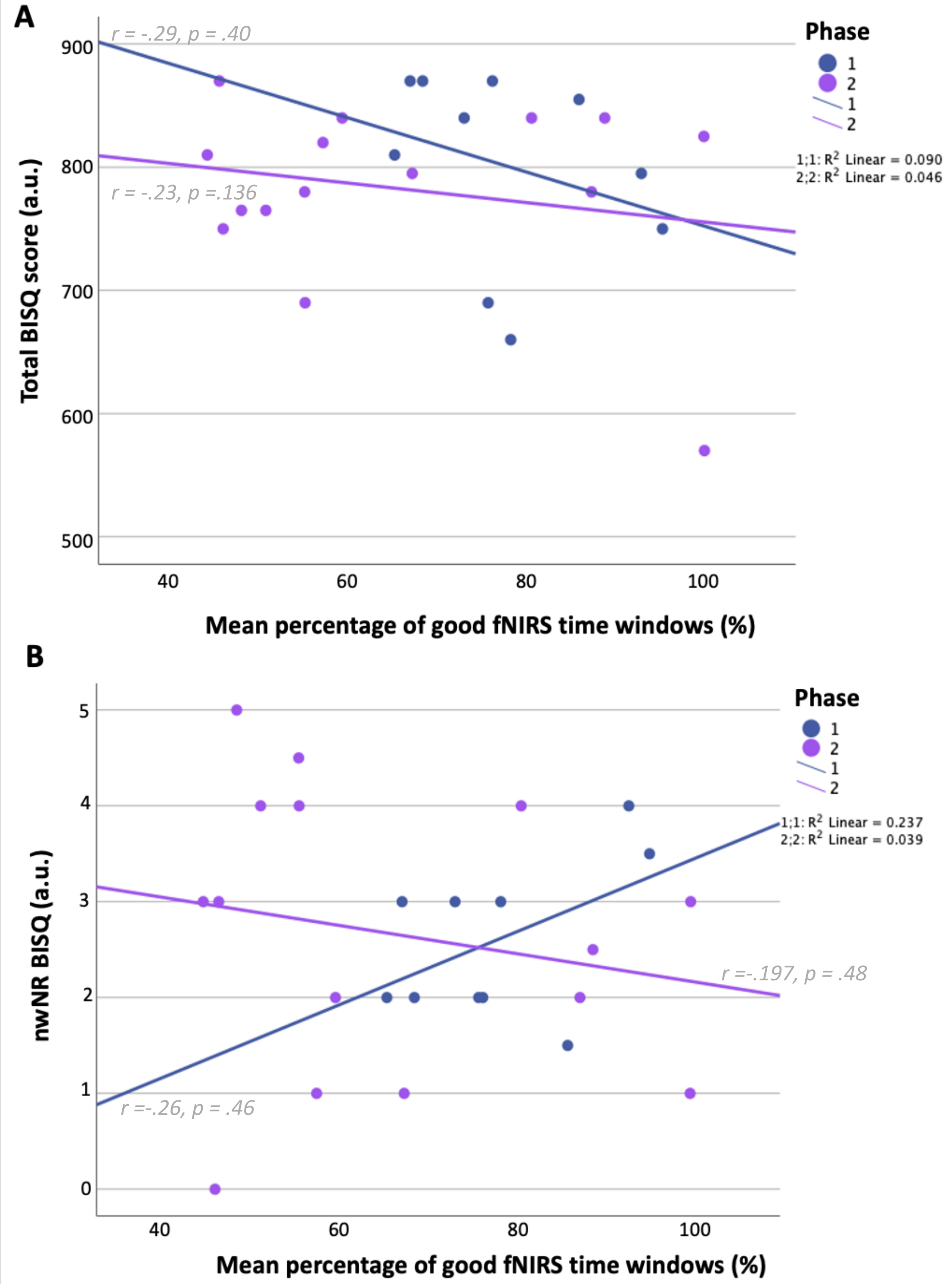

Figure S1. Scatterplot of mean percentage of good fNIRS time windows and the total sleep duration as measured by the Brief Infant Sleep Questionnaire (BISQ; A measure of habitual infant sleep) (A) and Night Waking Number as measured by the BISQ (B). *Note.* The correlations are not significant.

Table S1: Habitual sleep data

	Study phase	N	Mean	Std. Deviation
Night sleep duration	1	10	625.50	41.261
(in minutes)	2	15	629.67	66.614
Day sleep duration	1	10	175.50	87.764
(in minutes)	2	15	153.00	36.878
Total sleep duration	1	10	801.00	77.201
(in minutes)	2	15	782.67	74.207
Night waking number	1	10	2.600	.8097
	2	15	2.667	1.4840
Night waking duration	1	10	51.50	27.894
(in minutes)	2	15	42.33	33.105

There were no statistically significant differences in habitual sleep patterns between the infants who slept with either system.

SM Section 3: EEG sleep data

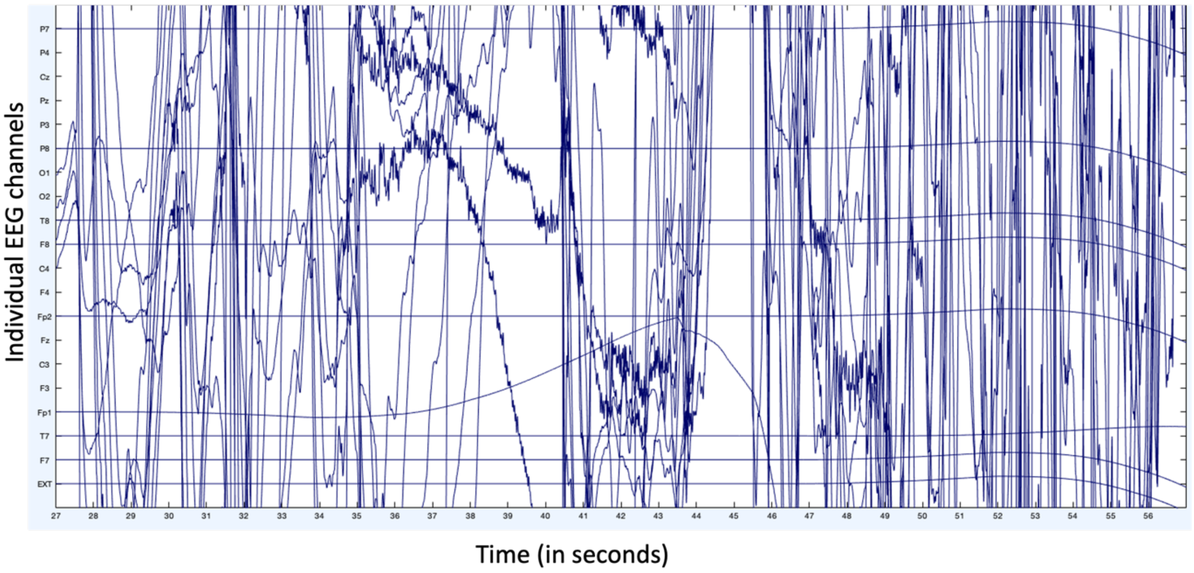

Figure S2. Illustration of poor quality EEG data (Example: disconnected mastoid reference); 30 seconds time scale for sleep stage scoring.

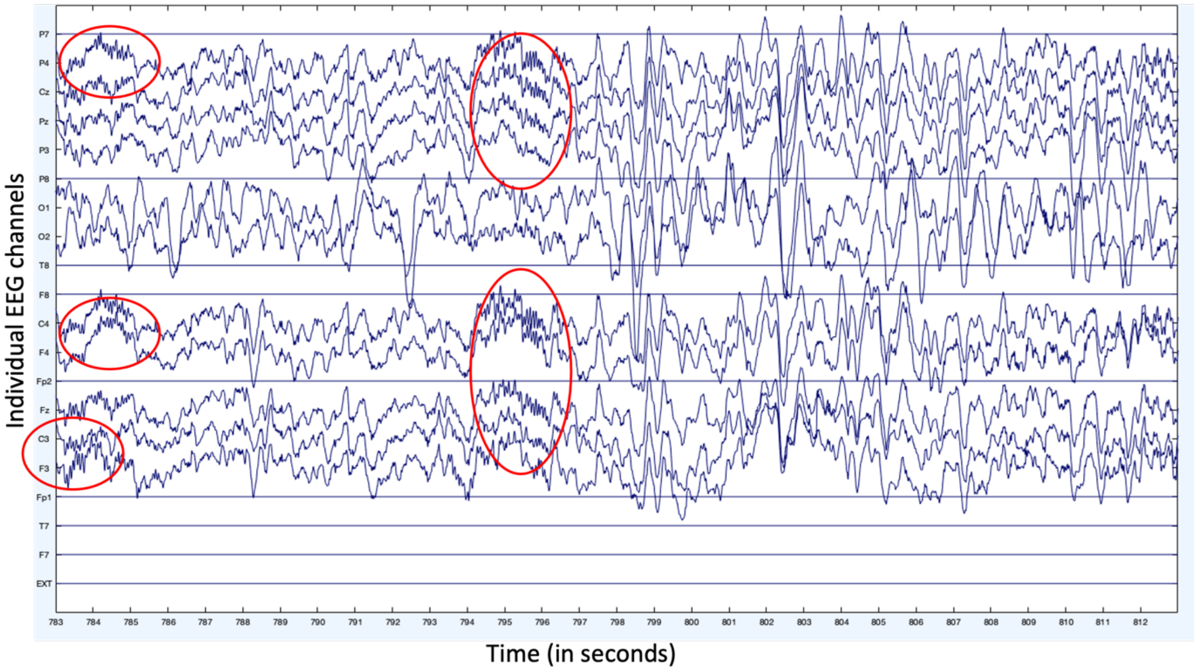

Figure S3. Illustration of good quality EEG data including sleep spindles

Section 4: Relating sleep EEG to sleep fNIRS data – an example.

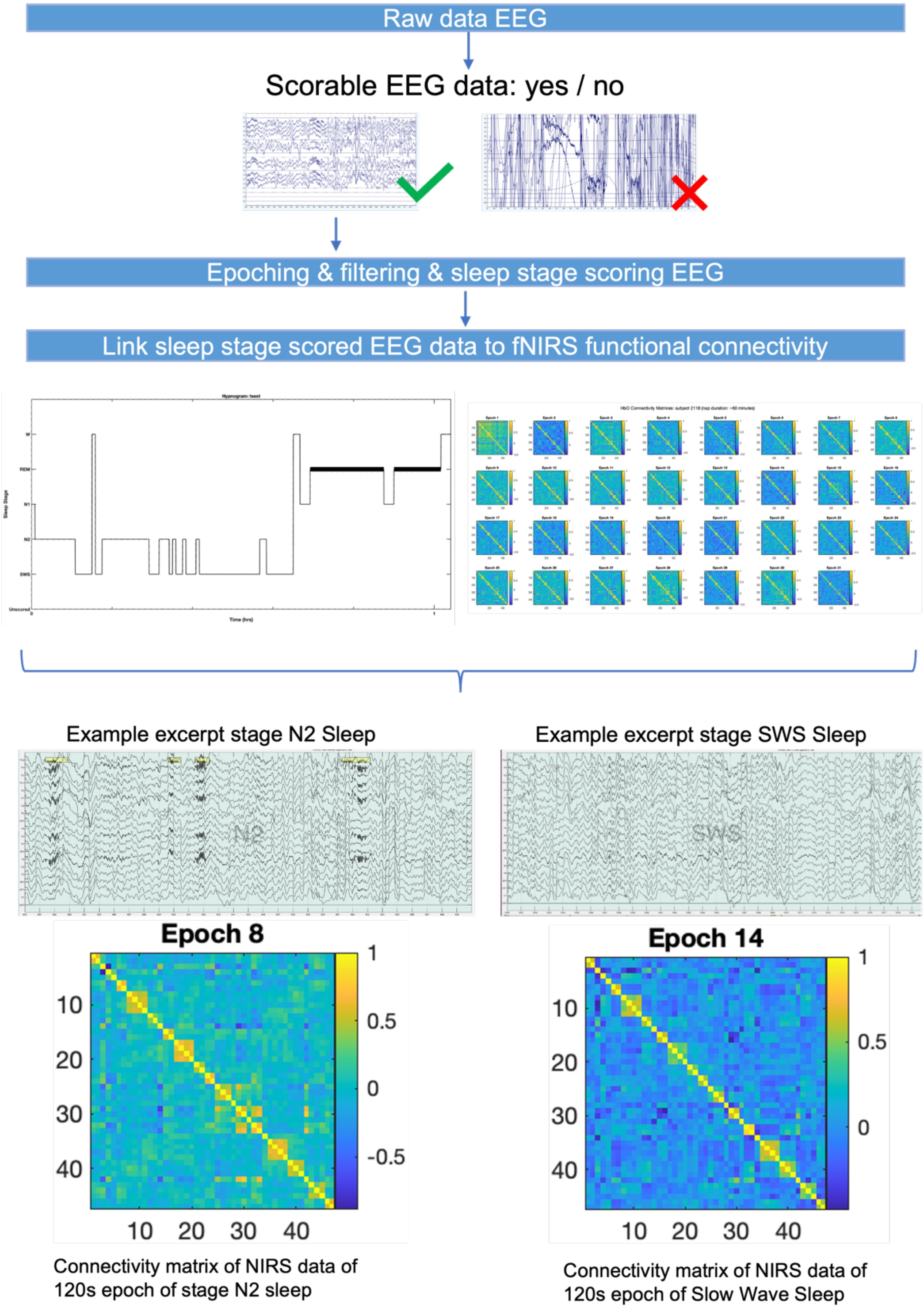

Figure S4. Illustration of how sleep staged scored EEG data could be linked to the fNIRS sleep data for one subject.

Section 5: Raw intensity plots of the fNIRS data for the single subject data presented in Figure 7

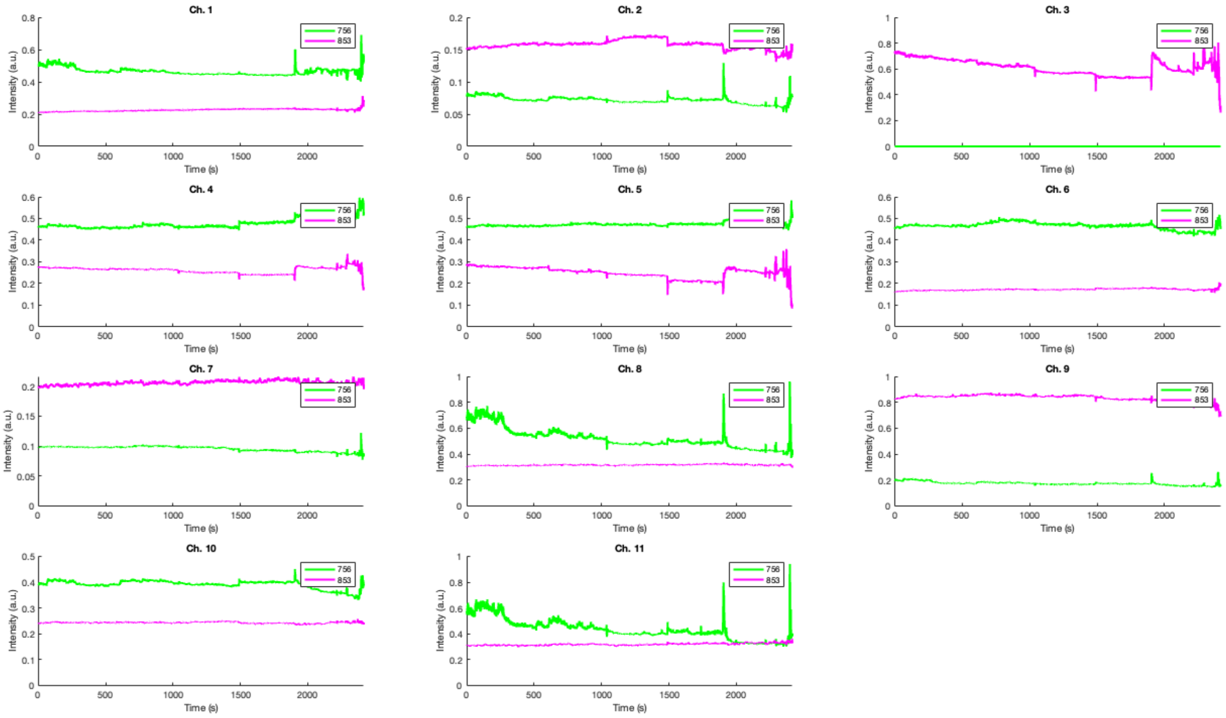

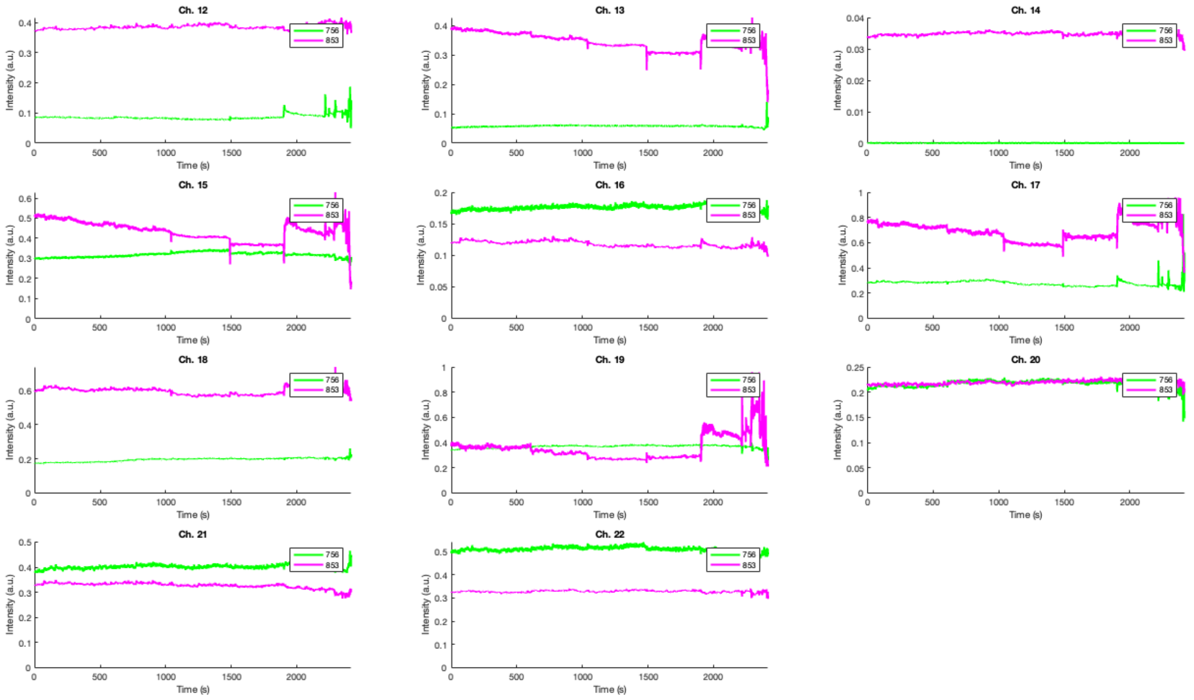

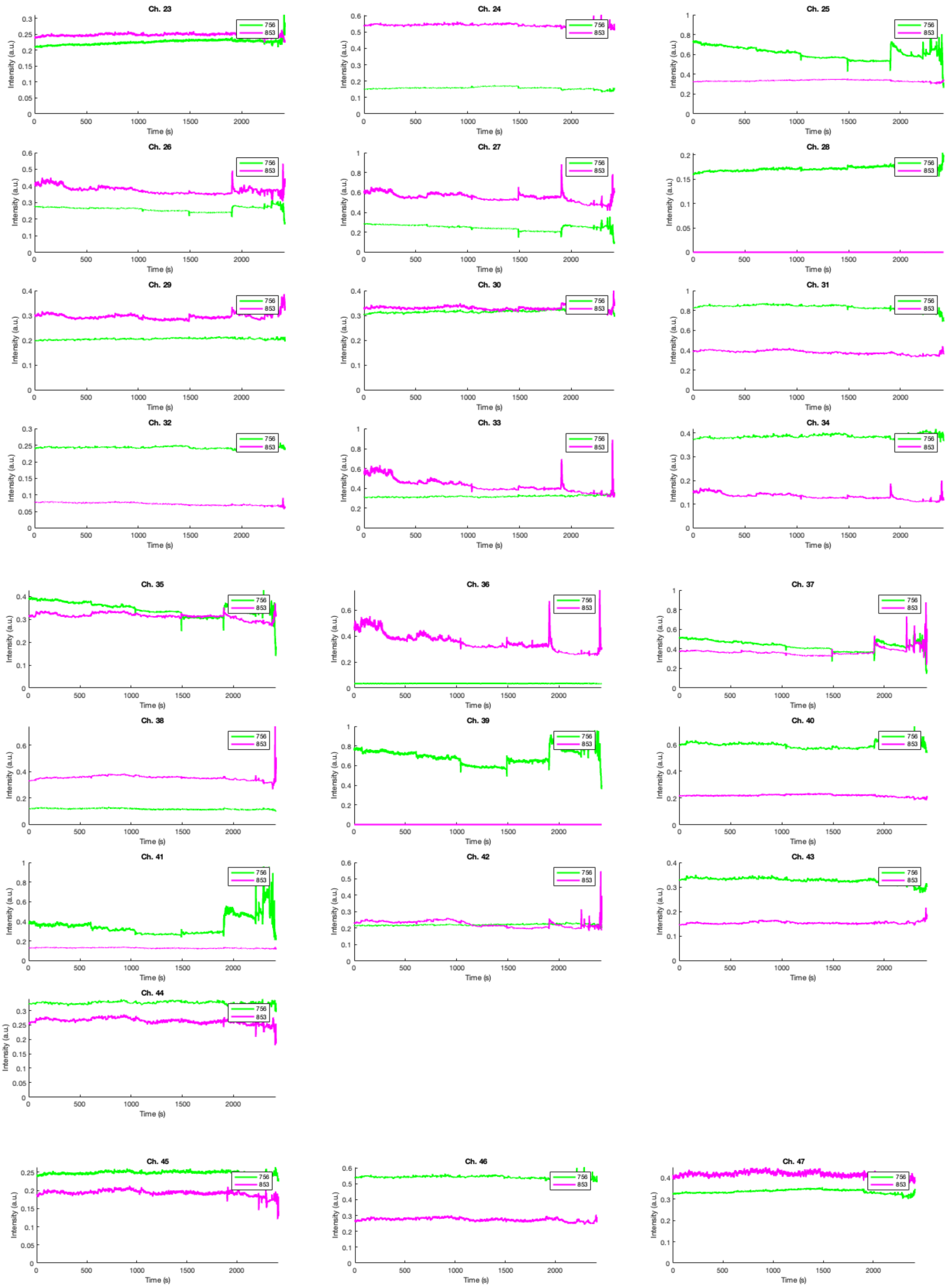

Figure S5. Raw intensity plots of all channels for single subject data of Figure 7. *Note.* This data is not trimmed to the exact behavioral nap length yet.
